# The specialized pro-resolving lipid mediator Protectin D1 affects macrophages differentiation and activity in Adult-onset Still’s disease and COVID-19, two hyperinflammatory diseases sharing similar transcriptomic profiles

**DOI:** 10.3389/fimmu.2023.1148268

**Published:** 2023-04-21

**Authors:** Luca Navarini, Marta Vomero, Damiano Currado, Onorina Berardicurti, Alice Biaggi, Annalisa Marino, Pietro Bearzi, Erika Corberi, Amelia Rigon, Luisa Arcarese, Alessandro Leuti, Marina Fava, Marta Fogolari, Alessia Mattei, Piero Ruscitti, Ilenia Di Cola, Federica Sambuco, Francesco Travaglino, Silvia Angeletti, Francesco Ursini, Erminia Mariani, Paola Cipriani, Felice Eugenio Agrò, Annamaria Iagnocco, Raffaele Antonelli Incalzi, Mauro Maccarrone, Roberto Giacomelli

**Affiliations:** ^1^ Clinical and Research Section of Rheumatology and Clinical Immunology, Fondazione Policlinico Universitario Campus Bio-Medico, Rome, Italy; ^2^ Rheumatology and Clinical Immunology, Department of Medicine, University of Rome “Campus Bio-Medico”, School of Medicine, Rome, Italy; ^3^ Neurochemistry of Lipids Unit, European Center for Brain Research, IRCCS Santa Lucia Foundation, Rome, Italy; ^4^ Department of Medicine, Campus Bio-Medico University of Rome, Rome, Italy; ^5^ Operative Research Unit of Clinical Laboratory, Fondazione Policlinico Universitario Campus Bio-Medico, Rome, Italy; ^6^ Research Unit of Clinical Laboratory Science, Department of Medicine, University of Rome “Campus Biomedico”, Rome, Italy; ^7^ Operative Research Unit of Anaesthesia, Intensive Care and Pain Management, Fondazione Policiclinico Campus Biomedico, Rome, Italy; ^8^ Rheumatology Unit, Department of Biotechnological and Applied Clinical Sciences, University of L’Aquila, L’Aquila, Italy; ^9^ Emergency Department, Fondazione Policlinico Universitario Campus Bio-Medico, Roma, Italy; ^10^ Medicine & Rheumatology Unit, IRCCS Istituto Ortopedico Rizzoli, Bologna, Italy; ^11^ Department of Biomedical and Neuromotor Sciences (DIBINEM), Alma Mater Studiorum University of Bologna, Bologna, Italy; ^12^ Medical and Surgical Sciences, Alma Mater Studiorum University of Bologna, Bologna, Italy; ^13^ Laboratory of Immunorheumatology and Tissue Regeneration, Istituto di Ricovero e Cura a Carattere Scientifico (IRCCS) Istituto Ortopedico Rizzoli, Bologna, Italy; ^14^ Research Unit of Anaesthesia, Intensive Care and Pain Management, Department of Medicine, Campus Bio-Medico University of Rome, Rome, Italy; ^15^ Academic Rheumatology Centre - AO Mauriziano Torino, Cattedra di Reumatologia - Dipartimento Scienze Cliniche e Biologiche, Università degli Studi di Torino, Turin, Italy; ^16^ Unit of Geriatrics, University of Rome “Campus Biomedico”, Rome, Italy; ^17^ Internal Medicine, Fondazione Policlinico Campus Biomedico, Rome, Italy; ^18^ Department of Biotechnological and Applied Clinical Sciences, University of L’Aquila, L’Aquila, Italy

**Keywords:** Adult-onset Still’s Disease, COVID-19, specialized pro-resolving mediators (SPMs), protectin D1, hyperinflammation

## Abstract

**Introduction:**

COVID-19 and autoinflammatory diseases, such as Adult-onset Still’s Disease (AOSD), are characterized by hyperinflammation, in which it is observed massive production and uncontrolled secretion of pro-inflammatory cytokines. The specialized pro-resolving lipid mediators (SPMs) family is one the most important processes counteracting hyperinflammation inducing tissue repair and homeostasis restoration. Among SPMs, Protectin D1 (PD1) is able to exert antiviral features, at least in animal models. The aim of this study was to compare the transcriptome of peripheral blood mononuclear cells (PBMCs) from patients with AOSD and COVID-19 and to evaluate the role of PD1 on those diseases, especially in modulating macrophages polarization.

**Methods:**

This study enrolled patients with AOSD, COVID-19, and healthy donors HDs, undergoing clinical assessment and blood sample collection. Next-generation deep sequencing was performed to identify differences in PBMCs transcripts profiles. Plasma levels of PD1 were assessed by commercial ELISA kits. Monocyte-derived macrophages were polarized into M1 and M2 phenotypes. We analyzed the effect of PD1 on macrophages differentiation. At 10 days, macrophages were analyzed for surface expression of subtypes markers by flow cytometry. Cytokines production was measured in supernatants by Bio-Plex Assays.

**Results:**

In the transcriptomes from AOSD patients and COVID-19 patients, genes involved in inflammation, lipid catabolism, and monocytes activation were specifically dysregulated in AOSD and COVID-19 patients when compared to HDs. Patients affected by COVID-19, hospitalized in intensive care unit (ICU), showed higher levels of PD1 when compared to not-ICU hospitalized patients and HDs (ICU COVID-19 vs not-ICU COVID-19, p= 0.02; HDs vs ICU COVID-19, p= 0.0006). PD1 levels were increased in AOSD patients with SS ≥1 compared to patients with SS=0 (p=0.028) and HDs (p=0.048). *In vitro* treatment with PD1 of monocytes-derived macrophages from AOSD and COVID-19 patients induced a significant increase of M2 polarization vs control (p<0.05). Furthermore, a significant release of IL-10 and MIP-1β from M2 macrophages was observed when compared to controls (p<0.05).

**Discussion:**

PD1 is able to induce pro-resolutory programs in both AOSD and COVID-19 increasing M2 polarization and inducing their activity. In particular, PD1-treated M2 macrophages from AOSD and COVID-19 patients increased the production of IL-10 and enhanced homeostatic restoration through MIP-1β production.

## 1 Introduction

Hyperinflammation is a common trait in many different diseases including, from one hand, monogenetic autoinflammatory diseases and, on the other hand, diseases in which this hyperactivity may be polygenic, such as systemic autoimmune diseases, Adult-onset Still’s Diseases (AOSD), as well as Behçet disease (1). The pandemic of COVID-19, acronym of the English COronaVIrus Disease 19, caused by SARS-CoV-2, a disease characterized by serious pulmonary and systemic complications, offered us a new a post-viral model of acute hyperinflammation, characterized by a massive production and uncontrolled secretion of pro-inflammatory cytokines which, together with the local release of other mediators, are responsible for the development of severe acute disease (2). Hyperactivated monocytes and macrophages have been found in COVID-19 patients and this amount correlates with a more severe form (3). This uncontrolled systemic inflammation strongly links COVID-19 with a specific polygenic autoinflammatory disease, such as AOSD, and, in fact, many drugs commonly used for the treatment of AOSD have been also approved for COVID-19 disease, such as the IL-1 inhibitor Anakinra (4–6). AOSD is a systemic inflammatory disease involving predominantly young adults and inducing a broad spectrum of clinical manifestations, including fever, arthritis, skin rash, splenomegaly, and enlarg1ement of lymph nodes (7). Both COVID-19 and AOSD are characterized by common inflammatory pathways, macrophages shift from anti-inflammatory M2 to pro-inflammatory M1, and the production of pro-inflammatory mediators, including IL-6, IL-1β, and TNF‐α, to modulate the local immune responses sometimes developing to the so-called “cytokine storm” syndrome (8, 9). As previously reported, severe forms of COVID-19 and AOSD shared similar clinical and serological features including fever, a common lung involvement, and increased ferritin levels (10, 11). The shutdown of the local and systemic inflammatory response in these related conditions may be an innovative and effective therapeutic approach for their treatment. In fact, although acute inflammation represents a host defensive mechanism, an uncontrolled inflammation, as observed in these diseases, is the main factor leading to tissue injury and systemic illness. A better understanding of the key pathways related to control inflammation and its effects may ne theoretically considered a potential therapeutic target to counteract the damages. It is well known that during inflammation, together with the release of pro-inflammatory molecules, the biosynthesis of specialized pro-resolving mediators (SPMs), which should ensure both the inflammatory shutdown and the repair of damaged tissues, is observed (12). This molecular family, including lipoxins (LX), resolvins (Rv), protectins (PD), and maresins (MaR), try to control inflammation and tissue homeostasis, by limiting the recruitment of neutrophils and stimulating the clearance of apoptotic cells (13). A failure in this mechanism may underlie persistent inflammation, as observed in infections and chronic diseases. Among the SPMs, the reduction of protectin D1 (PD1) levels has been found in the lungs of mice infected with the H5N1 influenza virus and the treatment with PD1 increased animal survival (14). Moreover, PD1 is able to decrease the production of IL-1β, a cytokine which plays a pivotal role both in AOSD and COVID-19, from macrophages derived from a mouse model (15–17). Furthermore, the analysis of SPMs profile in synovial fluid obtained from patients with severe rheumatoid arthritis showed that PD1 levels were significantly higher in the synovial fluids of these patients when compared with osteoarthritis patients (18).

Taking together these data concerning the presence of PD1 in both infectious and arthritic autoimmune, characterized by high levels of inflammation, our work was aimed to understand the possible role of PD1 in modulating the common hyper-inflammatory pathways characterizing both AOSD and COVID-19. The definition of the specific genetic, molecular, and functional features of PD1 in both these diseases, characterized by hyperinflammation, will allow us to define new possible targets for specific treatments aimed to slow down the pathologic, uncontrolled hyperinflammation.

## 2 Materials and methods

### 2.1 Patients recruitment

Participants affected by COVID-19, AOSD, and healthy donors HDs were consecutively recruited at the Fondazione Policlinico Universitario Campus Bio-Medico, Rome, Italy. All the participants filled in and signed the informed consent. The study protocol was approved by the Internal Review Board (IRB) of the Fondazione Policlinico Universitario Campus Bio-Medico, Rome (Italy), protocol number 5416 oss. The study was conducted in compliance with the International Conference on Harmonization Good Clinical Practice guidelines and the Declaration of Helsinki. Key inclusion criteria for all the participants were: age>18 years, signed informed consent. In addition, all the participants with COVID-19 were positive for SARS-CoV-2 nasal-pharyngeal swab in the two days before the enrolment. Moreover, all the participants with AOSD were diagnosed according to Yamaguchi’s criteria. Key exclusion criteria were: (i) diagnosis of cancer at enrolment; (ii) infectious disease other than COVID-19 at the enrolment; (iii) treatment >0.5 mg/kg/day of prednisone (or equivalent) at the enrolment as well as in the last seven days, because it has been recently showed that high dosages of dexamethasone upregulate the production of SPMs (19, 20); (iv) pregnancy or lactation; (v) only for COVID-19 participants, symptom onset more than five days before the enrolment; (vi) diagnosis of other autoimmune or autoinflammatory diseases; (vii) diagnosis of congestive heart failure; (viii) diagnosis of chronic obstructive pulmonary disease. All the participants underwent clinical assessment and blood sample collection. Patients with COVID-19 were further stratified in patients who were hospitalized in Intensive Care Unit (ICU) or not (Non-ICU). Patients with AOSD were stratified according to presence or not of multiorgan involvement, which is an independent risk factor of poor prognosis (21, 22), based on systemic score proposed by Pouchot et al. in (SS)=0 or SS≥1 (23).

### 2.2 Analysis of PD1 plasma levels

Levels of PD1 in the plasma of all participants and HDs were assessed by using Protectin D1 ELISA Kit (Cat#EKF58060, Biomatik) according to manufacturer’s instructions. Absorbance was measured at 450 nm by Infinite 200 PRO microplate reader (Tecan).

### 2.3 Monocytes-derived macrophages differentiation and treatment

Peripheral blood mononuclear cells (PBMCs) from participants and HD were obtained by using Ficoll-Paque. PBMCs were left overnight in RPMI 1640 supplemented with 10% FBS, 1% L-glutamin, 1% Na-pyruvate, and 1% penicillin/streptomycin in human serum-coated dishes for monocytes adhesion. Successively, non-adherent cells were removed by washing, and adherent monocytes were gently rinsed with PBS and cultured in fresh complete medium, supplemented with 50 ng/mL of M-CSF for 8 days, changing the medium every 2 days. At day 8, cells were differentiated in M1 macrophages by using IFN-gamma (10 ng/ml) and LPS (100 ng/ml), and in M2 macrophages by using IL-4 (20 ng/ml) for 2 more days. To analyze the effect of PD1 on macrophages differentiation, PD1 (10ng/ml) was added every day in half of the flasks. To find the correct dosage of PD1 in our experimental setting, a dose-response curve was obtained in cells from HDs. At day 10, adherent M1 and M2 macrophages were collected and analyzed for immunophenotyping and biomolecular assays. Supernatants were collected and stored at -80°C until cytokines, chemokines and Resolvin D1 dosages.

### 2.4 Flow cytometry

After *in vitro* treatment with PD1, monocytes-derived macrophages were analyzed for the expression of specific markers (CD68 for the whole cells, CD80 for M1 phenotype, and CD206 for M2 phenotype) by flow cytometry using CytoFLEX cytometer (Beckman Coulter). Briefly, macrophages were washed in Ca2+/Mg2+ -free PBS and stained with fluorophore-conjugated antibodies against CD68 (APC; Miltenyi Biotec), CD80 (PE; Miltenyi Biotec), CD206 (FITC; Miltenyi Biotec) and IgG isotype control antibodies for 30 min at +4°C. For each sample, at least 50,000 events were acquired. The expression of CD80 and CD206 surface markers was analyzed by gating cells on CD68+ macrophages using CytExpert software (Beckman Coulter) and was evaluated by Mean fluorescence intensity (MFI, **Supplementary Figure 1A**). As shown in **Supplementary Figure 2B**, macrophages from COVID-19 and AOSD patients polarized toward M1 phenotype showed an increased expression of M1 marker CD80, while cells polarized to M2-like macrophages showed an increased expression of M2 marker CD206.

### 2.5 Analysis of cytokine release

The release of G-CSF, GM-CSF, IFN-γ, IL-1β, IL-2, IL-4, IL-5, IL-6, IL-7, IL-8, IL-10, IL-12 (p70), IL-13, IL-17A, MCP-1 (MCAF), MIP-1β, TNF-α from M1 and M2 macrophages was analyzed in duplicate by using Bio-Plex Pro Human Cytokine 17-plex Assay kit (Bio-rad) following manufacturer’s instruction. The amounts of cytokines and chemokines in supernatants (pg/mL) were measured on Bioplex 200 system and data were analysed using the Bio-Plex Manager software version 6.0 (Bio-Rad Laboratories, Hercules, CA, USA). Standard levels between 70 and 130% of the expected values were considered to be accurate and were used. In general, at least 6 standards were accepted and used to establish standard curves following a 5 parameter logistic regression model (5PL).

### 2.6 RNA sequencing of PBMCs isolated from COVID-19 and AOSD patients

Total RNA was extracted from PBMCs by using all prep DNA/RNA/miRNA universal kit (Qiagen, Germany). Extracted RNA was submitted to a biotech company (GALSEQ srl, *Via* Vincenzo Monti, 8 - 20123 Milano) for a bulky RNA sequencing, including total RNA sample detection, mRNA enrichment, synthesis of double-stranded cDNAs, end repair/dA-tailing module, fragment selection, and PCR amplification, library detection, and Illumina sequencing. The method for screening differentially expressed genes among the 3 groups was filtered by P value < 0.05, and fold-change (FC) values were more than 1.2-fold. Heatmaps were generated in R statistical software (version 3.0.3; R Foundation for Statistical Computing, Vienna, Austria) with the NMF package.

### 2.7 Detection of SARS-CoV-2 in cell supernatants and pellet

PCR for SARS-CoV-2 was performed to assess the presence of the viral RNA in the cell supernatants from M2 macrophages of AOSD and of COVID-19 patients. Samples were extracted using Versant Sample Preparation 1.0 on Versan kPCR Molecular System SP followed by RT-PCR amplification on Quant Studio 5 by using Altona Real Start SARS-CoV2 RT-PCR Kit 1.0.

### 2.8 Statistical analysis

Continuous variables are expressed as median (25th – 75th percentile) or percentage, as appropriate. Normality of continuous variables has been assessed using the Shapiro-Wilk test, whereas differences between continuous variables have been analyzed using Wilcoxon test for paired data and Mann-Whitney test for unpaired data. Spearman test was used for correlation analysis. Statistical analysis was performed using GraphPad Prism 7 (GraphPad Software, Inc., San Diego, Ca, USA). The DEG between COVID19 and HCs and between AOSD and HCs were analyzed for their molecular pathways by the PANTHER (Protein ANalysis THrough Evolutionary Relationships) classification system. P values <0.05 were considered statistically significant.

## 3 Results

### 3.1 Clinical features COVID-19 and participants and HDs

Twenty-one COVID-19 participants were included in the study. The main demographic, anthropometric and clinical characteristics of this study population are reported in **Table 1**.

**Table 1:** COVID-19 patients characteristics.

	COVID-19 patients (n=21)	Non-ICU COVID-19 (n=11)	ICU COVID-19 (n=10)	p
Deaths, n (%)	0 (0)	0 (0)	0 (0)	
Male, n (%)	15 (71.43)	7 (63.64)	8 (80)	0.407
Hospitalization length, days (median, 25-75 pctl)	18.5 (8.5-26.5)	12 (5-23)	23 (19-51)	0.05
Age, years (median, 25-75 pctl)	58 (48-65)	58 (44-66)	58 (50-65)	0.6
WBC, n (median, 25-75 pctl)	10450 (9320-12030)	9840 (9140-11590)	11745 (9830-14250)	0.1
Neutrophils, n (median, 25-75 pctl)	9340 (7450-10490)	8590 (7450-9710)	10505 (7420-12710)	0.09
Lymphocytes, n (median, 25-75 pctl)	930 (650-1180)	990 (680-1210)	750 (540-1140)	0.2
Hb, g/dL (median, 25-75 pctl)	12.6 (11.9-13.2)	12 (11.7-13.2)	12.8 (12.5-13.7)	0.1
MCV, fL (median, 25-75 pctl)	87.7 (83.8-92.8)	89 (83.4-92.8)	87.5 (86.2-89.3)	0.9
PLT, n (median, 25-75 pctl)	300100 (200000-353000)	260000 (211000-366000)	303550 (187000-343000)	0.5
CRP, mg/dL (median, 25-75 pctl)	8.4 (3.07-13.94)	4.33 (0.92-11.78)	10.525 (6.58-15.84)	0.1
Ferritin, ng/mL (median, 25-75 pctl)	793 (181-2128)	184.5 (132.5-407.5)	1959.5 (1094-2669)	0.006
LDH, U/L (median, 25-75 pctl)	537 (447-583.5)	452.5 (374-531)	544.5 (520-621)	0.3
Glycemia, mg/dL (median, 25-75 pctl)	106 (100-151)	104.5 (100-151)	125.5 (104.5-205)	0.3
BUN, mg/dL (median, 25-75 pctl)	41.95 (34-62)	45 (33-66.5)	39.65 (35-57.6)	0.4
Creatinine, mg/dL (median, 25-75 pctl)	0.69 (0.55-0.89)	0.77 (0.65-0.94)	0.67 (0.53-0.8)	0.1
AST, U/L (median, 25-75 pctl)	31 (26-48)	31 (26-45)	36.5 (28-73)	0.5
ALT, U/L (median, 25-75 pctl)	30 (19-69)	20 (15-82)	30.5 (20-69)	0.3
Fibrinogen, mg/dL (median, 25-75 pctl)	646 (579-788)	579 (540-646)	734.5 (604-789)	0.1
PT, sec (median, 25-75 pctl)	14.85 (13.6-15.6)	13.6 (13.3-15.2)	15.6 (14.2-17.1)	0.1
PTT, sec (median, 25-75 pctl)	30 (27.7-36.4)	34.4 (29.1-42.7)	28.2 (26.3-36.4)	0.3

ICU, Intensive care unit; WBC, white blood cells; Hb, hemoglobin; MCV, mean corpuscular volume; PLT, platelets; CRP, C-reactive protein; BUN, blood urea nitrogen; LDH, Lactic Acid Dehydrogenase; AST, aspartate aminotransferase; ALT, alanine transaminase; PT, prothrombin time; PTT, partial thromboplastin time.

All the participants were hospitalized, with a large preponderance of male (71.43%) and a median age of 58 (48–65). Of note, we found statistically significant higher ferritin levels between ICU and non-ICU patients (1959.5 vs184.5, p=0.006), and higher PCT (0.2 vs 0.05, p=0.03). Participants who need ICU had a longer hospitalization (days in hospital) when compared to non-ICU participants (23 vs 12, p=0.05).

Five patients with AOSD, out of 13, had a systemic score ≥1. The most frequent manifestations at the time of collection were arthralgia (8/13), arthritis (5/13) and fever (3/13). In the SS ≥1 group of patients, at the time of collection, 3 patients were on glucocorticoid therapy and 2 patients were on biological treatment (one patient with tocilizumab and the other with anakinra), in all the other patients, when possible, the blood samples were collected during GC or biological drugs suspension or prior treatment. Moreover, we found statistically significant higher levels of ferritin and CRP in the SS ≥1 group as compared to SS=0 group. AOSD patients’ characteristics are summarized in **Table 2**. Moreover, we enrolled thirteen HDs age- and sex-matched to AOSD patients.

**Table 2:** AOSD patients characteristics.

	AOSD (n=13)	AOSD SS=0 (n=8)	AOSD ≥ 1 (n=5)	p
Age, years (median, 25-75 pctl)	50 (34-58)	50 (30-62)	48 (27.5-57.5)	0.59
Male, n (%)	8 (61.5)	4 (50)	4 (80)	0.3
Arthralgia, n (%)	8 (61.5)	4 (50)	4 (80)	0.3
Arthritis, n (%)	5 (38.5)	3 (37.5)	2 (40)	0.9
Fever, n (%)	3 (23.1)	0 (0)	3 (60)	0.06
Pleuritis, n (%)	0 (0)	0 (0)	0 (0)	–
Pneumonia, n (%)	0 (0)	0 (0)	0 (0)	–
Pericarditis, n (%)	0 (0)	0 (0)	0 (0)	–
Splenomegaly, n (%)	1 (7.7)	0 (0)	1 (20)	0.8
Lymphadenomegaly, n (%)	0 (0)	0 (0)	0 (0)	–
Leukocytosis, n (%)	2 (15.4)	0 (0)	2 (40)	0.3
Abdominal pain, n (%)	1 (7.7)	0 (0)	1 (20)	0.8
Pharyngodynia, n (%)	1 (7.7)	0 (0)	1 (20)	0.8
ESR, mm/h (median, 25-75 pctl)	8.5 (2.5-34.5)	5 (2-27.5)	65 (4.75-180)	0.16
CRP, mg/L (median, 25-75 pctl)	4.4 (2.3-13.75)	3.15 (1.35-7)	61.25 (8.15-123)	0.04
Ferritin, ng/mL (median, 25-75 pctl)	232 (102-621)	105 (43-348)	900 (621-1580)	0.016
BDMARDs, n (%) Anakinra, n (%) Canakinumab, n (%) Tocilizumab, n (%)	5 (38.5) 3 (23.1) 1 (7.7) 1 (7.7)	3 (37.5) 2 (25) 1 (12.5) 0 (0)	2 (40) 1 (20) 0 (0) 1 (20)	0.9
Methotrexate, n (%)	2 (15.4)	1 (12.5)	1 (20)	0.7
Glucocorticoids	8 (61.5)	6 (75)	2 (40)	0.2
Disease pattern: - monocyclic, n (%) - polycyclic, n (%) - chronic, n (%)	2 (15.4) 7 (53.8) 4 (30.8)	1 (12.5) 4 (50) 3 (37.5)	1 (20) 3 (60) 1 (20)	0.2

AOSD, Adult-onset Still’s disease; ESR, erythrocyte sedimentation rate; bDMARDs, biologic disease-modifying anti-rheumatic druds.

### 3.2 Monocytes-related pathways are activated in COVID-19 and AOSD patients

**Figure 1:**
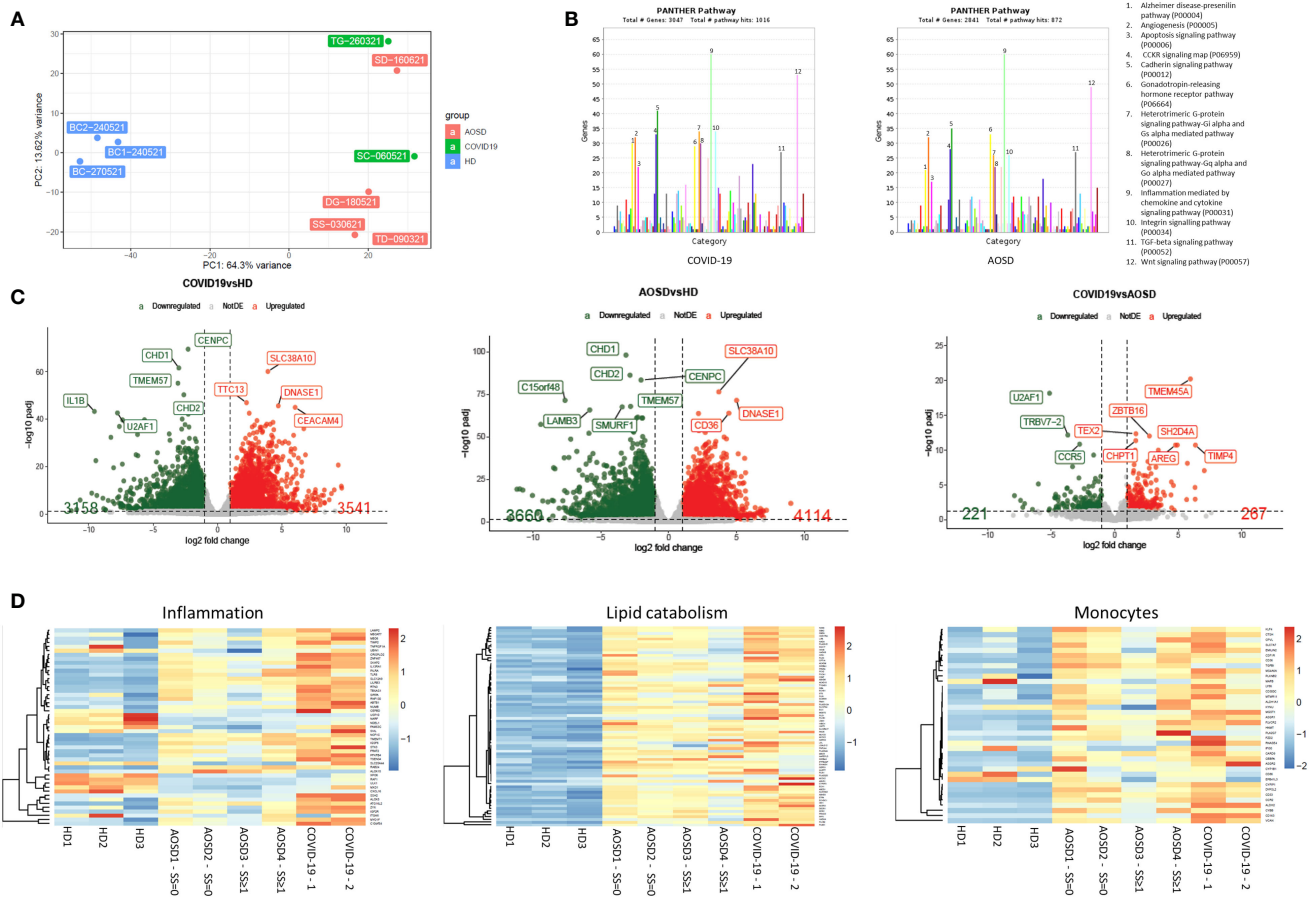
Transcriptome analysis of peripheral immune cells from HDs, COVID-19 and AOSD patients. **(A)** PCA plot. PCA shows the sample in the 2D plane spanned by their first two principal components. **(B)** Pathway analysis by Pantherdb.org. Bar chart of enriched pathways in COVID19 and AOSD patients. **(C)** Volcano plots. Volcano plot representation of differential expression analysis of genes in COVID-19 vs HD, AOSD vs HD, and COVID-19 vs AOSD. Red and green points mark the genes with significantly increased or decreased expression (FDR<0.05). The x-axis shows log2fold-changes in expression and the y-axis the log odds of a gene being differentially expressed. **(D)** Transcriptome analysis from 3 HDs, 2 COVID-19 patients and 3 AOSD patients of genes involved in inflammation, lipid catabolism and monocytes. HD, healthy donors; AOSD, Adult-onset Still’s disease.

Principal component analysis (PCA), using data from HDs, AOSD and COVID-19 patients, is plotted in **Figure 1A**. The plot shows the differences in PBMCs gene expression among studied groups. In the first principal component (accounting for 64% of variation), it is clearly evident the notable separation between HDs and the other two groups, with AOSD and COVID-19 patients located really close to each other. Furthermore, volcano plots show a general overview of the differential gene expression obtained in HDs, COVID-19, and AOSD patients (**Figure 1C**), where differentially expressed genes (DEGs) are highlighted in red (upregulated) and in green (downregulated) based on the p-value and FC variation among the three groups. Only 488 genes are significantly ((|log2 FC| ≥ 1 and FDR < 0.01)) deregulated among COVID-19 and AOSD patients. Of note, as reported on **Figure 1C**, same specific genes, in particular c*dh1, cdh2, cenpc, tmem57 (*downregulated), and *dnasi1, scl38a10* (upregulated) show the same expression in both COVID-19 and AOSD patients. These genes code for proteins involved in cell cycle, gene expression regulation and transmembrane transport. Panther analysis confirms the similarities between PBMCs RNAseq between COVID19 and AOSD patients (**Figure 1B**). There is an overlap in the enriched pathways between these patients. Of note, among these pathways are listed inflammation mediated by chemokine and cytokine signaling pathway, integrin signaling pathway, TGF-β signaling pathway, and Wnt signaling pathway. Transcriptomic heatmaps (**Figure 1D**) show that genes involved in inflammation and lipid catabolism are specifically dysregulated in COVID-19 and AOSD patients when compared to HDs. Moreover, genes associated with monocytes phenotype and function were upregulated in both COVID-19 and AOSD condition.

### 3.3 Levels of protectin D1 are increased in patients affected by COVID-19 and AOSD

**Figure 2:**
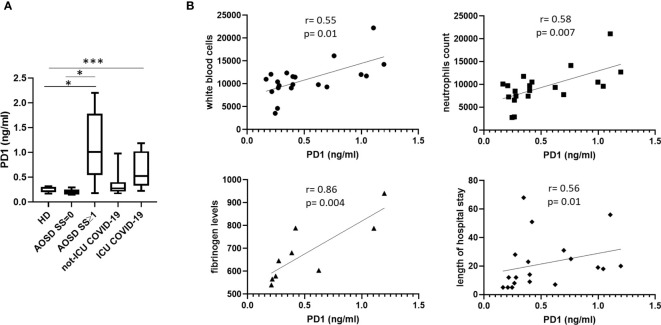
Levels of protectin D1 in HDs, COVID-19 and AOSD patients. **(A)**. Plasma levels of PD1 (ng/ml) in HDs (n=13) and patients affected by COVID-19 (not-ICU COVID-19, n= 11; ICU COVID-19, n=10) and AOSD (n=13) patients. Data are represented as box plots displaying medians, 25th and 75th percentiles as boxes, and 10th and 90th percentiles as whiskers. HD vs AOSD SS≥1, *p= 0.04; AOSD SS=0 vs AOSD SS≥1, *p=0.02; HD vs ICU COVID-19, p= 0.0006; not-ICU COVID-19 vs ICU COVID-19, *p= 0.02, *** p < 0.0001 **(B)** Correlation analysis between PD1 levels and white blood cells count, neutrophils count, fibrinogen levels and length of hospital stay in patients with COVID-19. Spearman correlation and linear regression coefficients are displayed. ICU, intensive care unit.

Patients affected by COVID-19, hospitalized in intensive care unit (ICU), showed higher levels of PD1 when compared to not-ICU hospitalized patients and HDs (ICU COVID-19 vs not-ICU COVID-19, p= 0.02; HDs vs ICU COVID-19, p= 0.0006 **Figure 2A**). Furthermore, in COVID-19 group, a direct correlation between PD1 levels and white blood cells count, neutrophilia, fibrinogen levels and length of hospital stay were found (**Figure 2B**). Similarly, PD1 levels were increased in AOSD patients with SS ≥1 compared to patients with SS=0 (p=0.02) and HDs (p=0.04, **Figure 2A**).

### 3.4 Effect of protectin D1 on monocytes-derived macrophages

**Figure 3:**
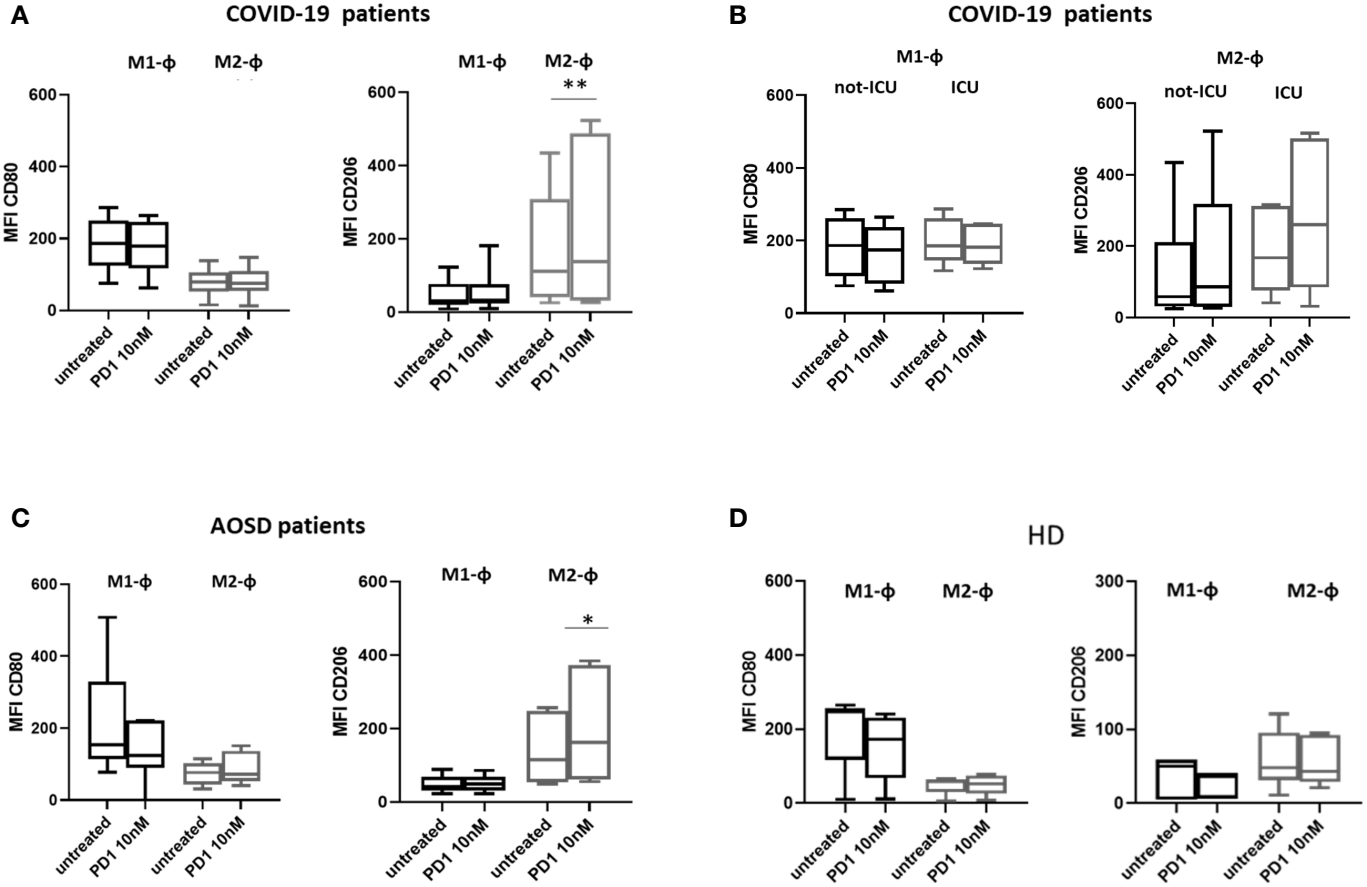
Effect of PD1 on polarization of macrophages from HDs, COVID-19 and AOSD patients. Expression of surface markers by flow cytometry on M1 and M2 macrophages from COVID-19 patients **(A)**, not-ICU and ICU COVID-19 patients **(B)**, AOSD patients **(C)** and HDs **(D)** after treatment with PD1. Data are represented as box plots displaying medians, 25th and 75th percentiles as boxes, and 10th and 90th percentiles as whiskers, and reported as Mean fluorescent intensity (MFI). M2 macrophages from COVID-19 patients, untreated vs PD1 **p = 0.009; M2 macrophages from AOSD patients, untreated vs PD1 *p =0.02. ICU, intensive care unit.

PD1 stimulation increased the expression of CD206 in M2 macrophages from both COVID-19 and AOSD patients (COVID-19: untreated vs PD1, p=0.009; AOSD: untreated vs PD1 p= 0.023, **Figures 3A–C**). As far as COVID-19 patients are concerned, no statistically significant change in surface macrophages markers was found between not-ICU and ICU patients (**Figure 3B**). On the contrary, PD1 did not affect CD206 and CD80 expression both in M1 and M2 macrophages from HDs (**Figure 3D**).

**Figure 4:**
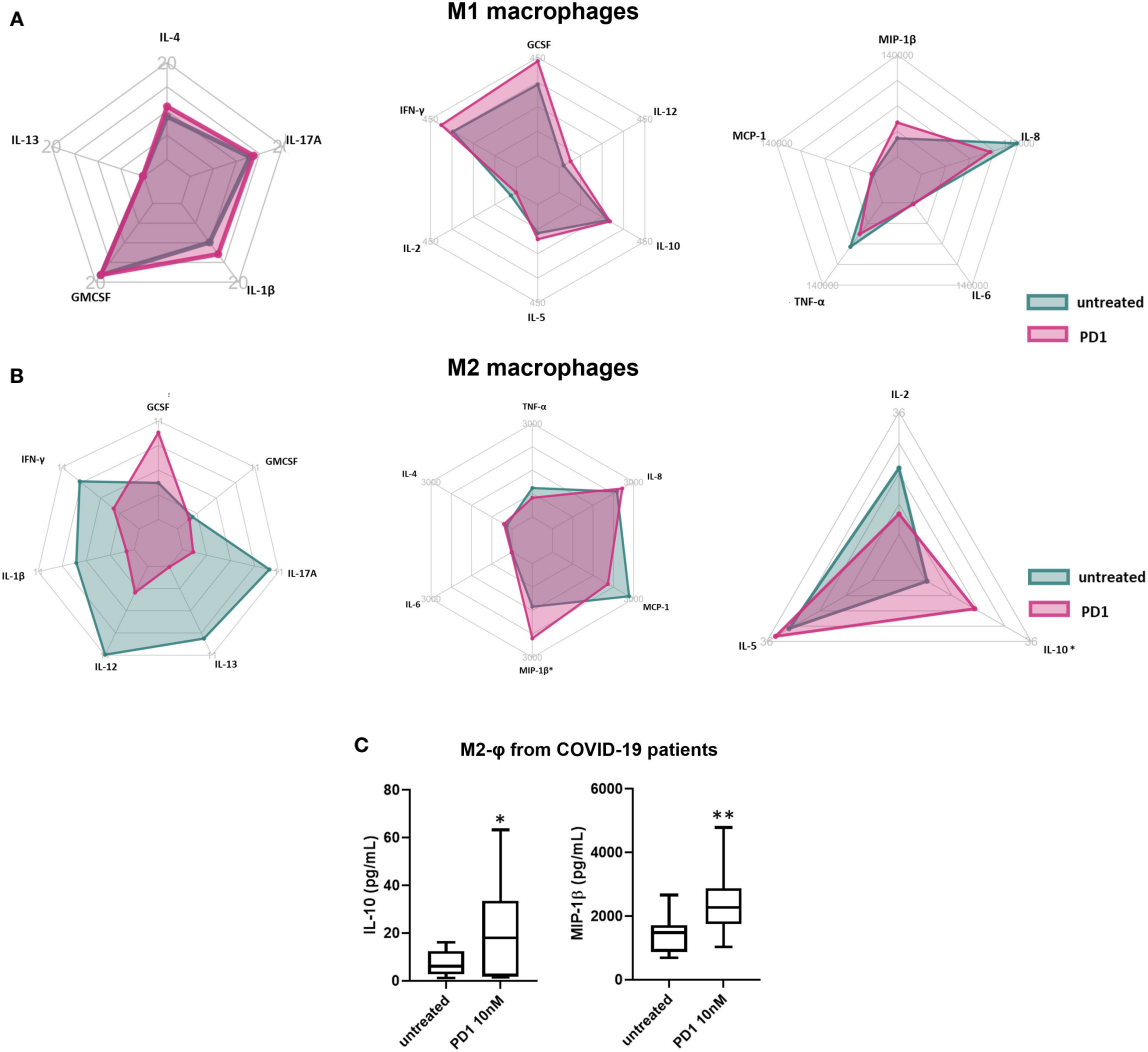
Effect of PD1 on secretion of cytokines/chemokines by macrophages from COVID-19 patients. Radar chart summarizing means of cytokines and chemokines profile of M1 **(A)** and M2 **(B)** macrophages from COVID-19 patients after *in vitro* treatment with PD1. Green plots represent untreated cells, while pink plot PD1-treated cells. **(C)** Multiplex analysis of IL-10 and MIP-1β levels in supernatants of M2 macrophages from COVID-19 patients treated with PD1. Data are reported as pg/mL and represent 12 independent experiments. IL-10, untreated vs PD1, *p= 0.03; MIP-1β, untreated vs PD1, **p= 0.003.

**Figure 5:**
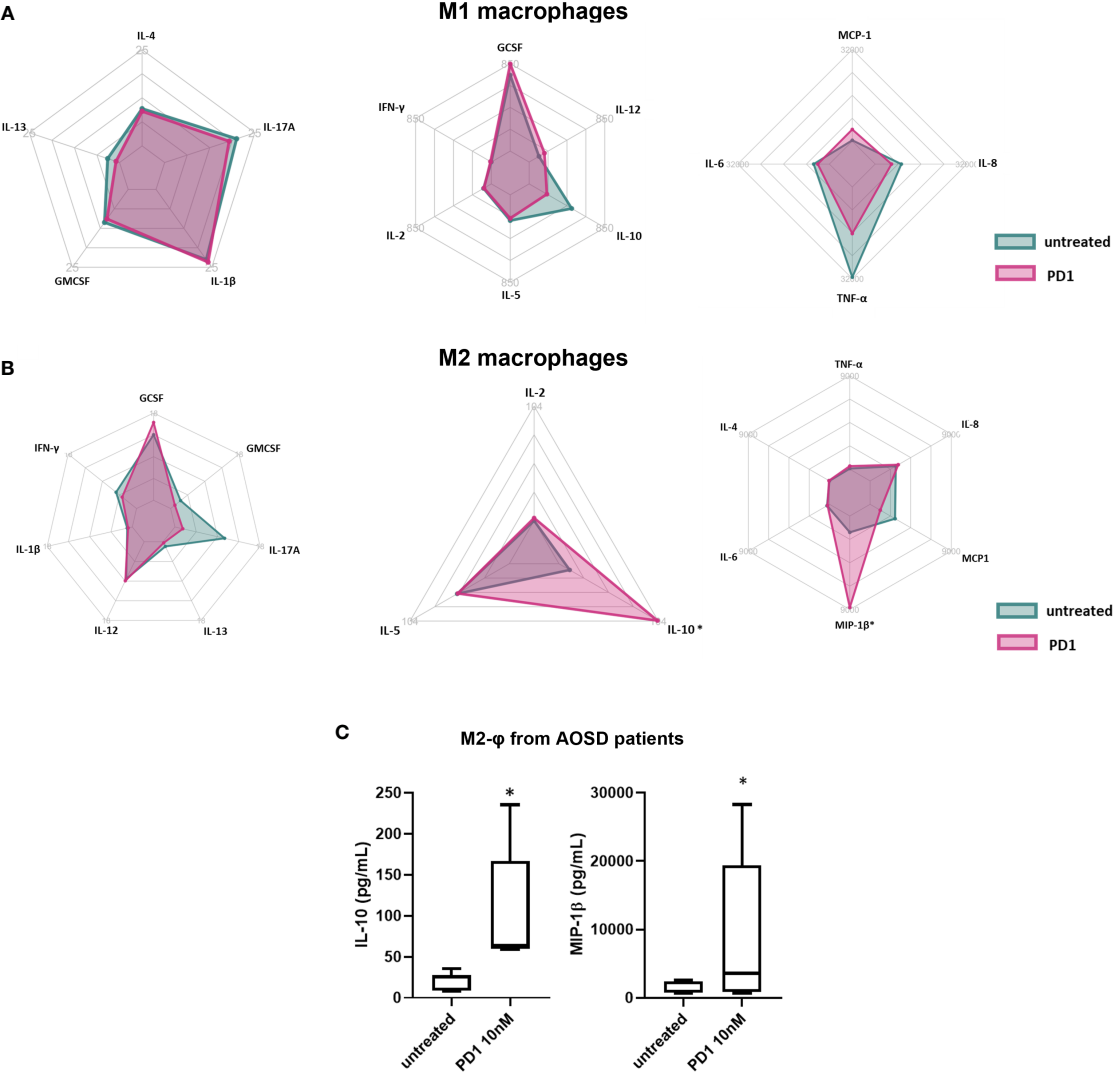
Effect of PD1 on secretion of cytokines/chemokines by macrophages from AOSD patients. Radar chart summarizing cytokines and chemokines profile of M1 **(A)** and M2 **(B)** macrophages from COVID-19 patients after in vitro treatment with PD1. Green plot represents untreated cells, while red plot PD1-treated cells. **(C)** Multiplex analysis of IL-10 and MIP-1β levels in supernatants of M2 macrophages from AOSD patients (n = 10) treated with PD1. Data are reported as pg/mL. IL-10, untreated vs PD1, *p= 0.03; MIP-1β, untreated vs PD1, *p= 0.03.

**Figures 4**, **5** shows the variation in cytokines and chemokines levels produced by macrophages obtained from COVID-19 and AOSD patients respectively treated or not with PD1. A significant increase of both IL-10 and macrophage inflammatory protein (MIP-1β) was observed after PD1 stimulation (IL-10, p= 0.03; MIP-1β, p= 0.003, **Figures 4B, C**). The same results were found in AOSD patients (IL-10, p= 0.03; MIP-1β, p= 0.03, **Figure 5C**). On the contrary, in macrophages from HDs, we did not observe any significant difference in secretion of cytokines and chemokines after PD1 stimulation (data not shown). Although not statically significant, the polar charts show a signal towards the reduction in the production of pro-inflammatory cytokines, and specifically IL-1β, IL-2, IL-12, IL-13, IL-17A secreted by M2 macrophages of COVID-19 patients, after PD1 stimulation (**Figure 4B**).

To confirm that the results of our experiments are not influenced by the presence of SARS-CoV-2, supernatants and pellets derived from cultured macrophages were subjected to Real-time PCR. As shown in **Supplementary Figure 4**, SARS-CoV-2 was not found in all the samples analyzed.

**Figure 6:**
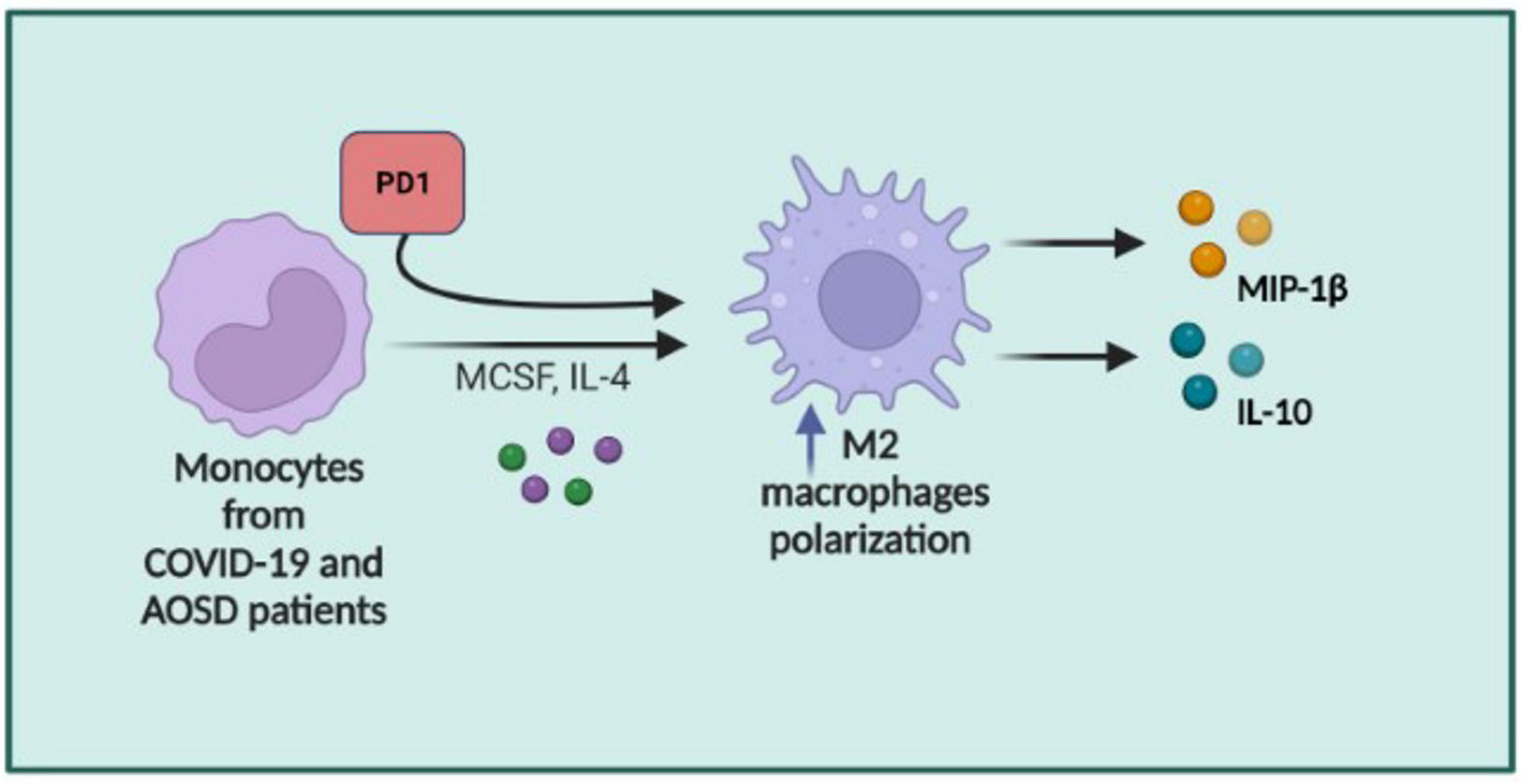
Summary of the in vitro experiments. *In vitro* PD1 induced the differentiation of monocytes from patients with both AOSD and COVID-19 in M2-macrophages and their secretion of IL-10 and MIP-1β (Created with BioRender.com).

Results of in vitro experiments are summarized in **Figure 6**.

## 4 Discussion

In this work, a significant overlapping in peripheral blood gene expression of both COVID-19 and AOSD patients, regarding inflammation, lipid catabolism, as well as monocytes activation pathways, was observed, supporting a strong similarity in the pathogenic steps of these two diseases, both characterized by innate immune response activation. These two conditions display common patterns of activated and silenced gene pathways, with only few hundred genes significantly differing in their expression. Our data point out the pivotal role of monocytes activation and subsequent inflammation in both COVID-19 and AOSD (24, 25). The majority of the overexpressed pathways may be modulated by bioactive lipids, for instance SPMs (26), which play a central role in diseases resolution process. Therefore, we assessed the presence and the function of PD1, a member of SPMs family, modulating innate immune responses and monocytes functions (27), especially during viral infections. Available literature reports that PD1 increases survival in a mouse model of H5N1 influenza virus infection (14), as well as improving a mouse model of kidney ischemia/reperfusion by inducing M2 phenotype in macrophages (28). Furthermore, in multiple sclerosis, this molecule was able to reduce monocyte activation, their migration through endothelia, as well as decreasing cytokine production (29, 30). We observed a close correlation between the levels of PD1 and the severity of COVID-19. Not surprisingly, ICU COVID-19 patients showed significant increase of PD1 when compared to not-ICU patients and HDs and our data mirror the results obtained in another genetic-correlated European cohort in which non-survivors COVID-19 patients showed increased circulating levels of PD1n-3 DPA (31). Furthermore, PD1 levels also correlated with some specific biomarkers of innate inflammation, such as neutrophilia and hyperfibrinogenemia, confirming the association between high levels of inflammation and production of PD1. As far as AOSD patients are concerned, similar results were observed between PD1 levels and the severity of the disease. Our results partially mirror what observed in COVID-19 patients, in which an increase of PD1 has been reported (31), while no data are available about AOSD and any molecule of the SPMs group. Surprisingly, we did not find any correlation between PD1 levels and ferritin. It is well known that both in COVID19 and in AOSD the levels of ferritin strongly correlate with disease the severity of the diseases , and also our study confirms the significance of this biomarker in this setting (32–35). Probably, the lack of a significant correlation between PD1 and ferritin might be referred to a relatively small sample size of our cohorts.

To better define the role of PD1 in these two hyperinflammatory diseases, we planned some functional experiments in which monocytes-derived macrophages were stimulated with the optimal dosage of PD1, assessed by a dose-response curve (**Figure 6**). We have chosen these cells, considering that in the peripheral blood of both COVID-19 and AOSD we observed a transcriptome profile toward monocytes activations (36).

Both in COVID19 and AOSD patients, we observed that adding PD1 in monocyte cultures, stimulated with M-CSF plus IFN-gamma plus LPS (to induce M1 polarization) or alternatively M-CSF plus IL-4 (to induce M2 polarization), a significant increase of CD206, a M2 macrophage marker whose expression is related to a downregulation of inflammatory processes (37, 38), was observed when compared to PD1 untreated condition. On the contrary, no difference was observed on CD80 expression between PD1-treated and PD1-untreated cells during M1 polarization. Our data clearly show that PD1 is able to potentiate the M2 polarization while it is unable to affect the M1 subset. Of note, when we analyzed the results obtained from monocytes-derived macrophages from HDs stimulated with PD1, we did not observe any significant effect of this molecule on M1 and/or M2 polarization, suggesting that an *in vivo* inflammatory stimulus may be present to induce a PD1-driven anti-inflammatory response.

Analyzing the cytokines production, in the cultured macrophages obtained from both Covid19 and AOSD patients, a significant increase of IL-10, after PD1 stimulation, was shown. IL-10 plays a main anti-inflammatory role during the immune response and its function has been confirmed also during COVID-19 (39). At present, our results seem to be the first observation of a direct link between PD1 and IL-10. Available literature reports that protectin DX (PDX, an isomer of PD1) increased the proportion of M2 macrophages in septic mice and in this model elevated levels of IL-10 were observed after PDX administration (40). Furthermore, the treatment of monocyte-derived macrophages with n–3 PUFA-rich lipid emulsion which is able to induce PDX release, significantly increased IL-10 secretion (41). Taking together these data may suggest that PD1 plays its anti-inflammatory effect *via* an increased release of IL-10. Interestingly, previous study showed that serum levels of IL-10 were higher in AOSD patients than in healthy controls and positively correlated with systemic score, suggesting its role in the suppression of pro-inflammatory cytokine in diseases characterized by cytokine storm (42–44).

Our data shows that PD1 was able also to induce the production of MIP-1β, also called CCL4, from M2 macrophages. MIP-1β is a chemokine that, after binding its receptor CCR5, induces the chemoattraction of different immune cells, including natural killer cells and monocytes (45). Again, our paper is the first demonstration that PD1 may enhance the production of MIP-1β from M2 macrophages. We may hypothesize that recruiting monocytes, *via* PD1/MIP-1β, in a milieu enriched of anti-inflammatory molecules such as IL-10, it could progressively increase the differentiation toward M2 macrophages of the recruited cells, thus contributing to switch off inflammation. Furthermore, MIP-1β, after stimulation with PD1, might stimulate homing of immune cells in order to clear cellular debris and thus contributing to homeostasis restoration (27).

Considering that the PD1 has been found frequently induced by virus infections, we searched the possible presence of SARS-CoV-2 RNA in the macrophages of COVID-19 patients. It is well known that SARS-CoV-2 may infect monocytes and macrophages (3) which expose on their surface the receptor for the virus, namely ACE2. Interestingly, we did not find virus RNA in our cells, thus confirming that our *in vitro* data mirror what may happen during a systemic or local inflammation, independently from viruses presence.

Some limitations of this study should be considered. Firstly, we enrolled only patients with COVID-19 who have been hospitalized and we did not recruit any outpatient. Therefore, we might have neglected some clinical and biological characteristics of patients with milder COVID-19 forms. In addition, we enrolled only patients with COVID-19 tested positive for SARS-CoV-2 nasal-pharyngeal swab in the two days before the enrolment and showing symptoms less than five days before the enrolment; as COVID-19 may worsen even after 5 days of infection, those patients cluster may be missed in this study (46). Moreover, all the patients with AOSD were taking glucocorticoid and/or immunosuppressants. Consequently, our data should be confirmed also in a population of newly diagnosed, treatment-free AOSD patients. Finally, we enrolled a relatively small number of patients, although the sample size was sufficient to define the statistically significance of our results.

Despite the aforementioned limitations, this study shows for the first time that the SPM PD1 is able to induce pro-resolutory programs in both AOSD and COVID-19, by enhancing M2 polarization and activity. In particular, M2 macrophages from AOSD and COVID-19 patients treated with PD1 were able to increase the production of the anti-inflammatory cytokine IL-10 and to enhance homeostatic restoration through MIP-1β production from phagocytes. Therefore, our data allow us to suggest PD1 pathway as a possible future therapeutic target in inflammatory diseases, such as AOSD and COVID-19.

## Data availability statement

The RNA sequencing data presented in the study are deposited in the BioProject respository, URL https://www.ncbi.nlm.nih.gov/bioproject/953515 and accession number PRJNA953515.

## Ethics statement

The studies involving human participants were reviewed and approved by Internal Review Board (IRB) of the Fondazione Policlinico Universitario Campus Bio-Medico, Rome (Italy). The patients/participants provided their written informed consent to participate in this study.

## Author contributions

LN: Conceptualization; Data curation; Formal analysis; Investigation; Supervision; Roles/Writing - original draft. MV: Conceptualization; Data curation; Investigation; Methodology; Roles/Writing - original draft. DC: Data curation; Investigation; Methodology; Roles/Writing - original draft. OB: Methodology; Software; Supervision; Visualization. AB: Investigation; Roles/Writing - original draft. AMar: Data curation; Formal analysis. PB: Data curation; Software. EC: Investigation; Methodology. AR: Investigation; Methodology. LA: Data curation. AL: Conceptualization; Data curation. MFa: Methodology. MFo: Methodology. AMat: Investigation. PR: Validation; Writing - review & editing. IC: Data curation; Software. FS: Investigation. FT: Investigation. SA: Methodology. FU: Validation; Writing - review & editing. EM: Investigation; Methodology. PC: Validation; Writing - review & editing. FA: Validation; Writing - review & editing. AI: Supervision. RA: Validation; Writing - review & editing. MM: Conceptualization; Validation; Writing - review & editing. RG: Conceptualization; Data curation; Supervision; Validation; Visualization; Writing - review & editing. All authors contributed to the article and approved the submitted version.

## Supplementary material

Supplementary Figure 1
**(A)** Gating strategy for flow cytometry analysis of surface markers in monocyte-derived macrophages. Population was first defined using forward scatter (FSC) and side scatter (SSC). Debris and apoptotic cells were excluded. The expression of surface markers CD206 and CD80 were analyzed on CD68-positive cells. **(B)** M1 and M2 Macrophages Polarization. Analysis of CD80 and CD206 expression, reported as mean fluorescence intensity, in monocyte-derived macrophages polarized towards M1 and M2 phenotype. MFI= mean fluorescence intensity.

Supplementary Figure 2 Cytokines and chemokines levels in supernatants of M1 **(A)** and M2 **(B)** macrophages from COVID-19 patients treated in vitro with PD1. Data are reported as pg/mL and represent 12 independent experiments.

Supplementary Figure 3 Cytokines and chemokines levels in supernatants of M1 **(A)** and M2 **(B)** macrophages from AOSD patients treated in vitro with PD1. Data are reported as pg/mL and represent 10 independent experiments.

Supplementary Figure 4 Representative assay amplification curves of SARS-CoV-2 RNA of one sample obtained from in vitro experiments on monocytes-derived macrophages from COVID-19 patients. Green line represents the amplification internal control, red line represents the amplification for SARS-CoV2 E (Envelope) gene while the blue line shows the amplification for SARS-CoV2 S (Spike) gene.
