# Supplementary figures and images for: The specialized pro-resolving lipid mediator Protectin D1 affects macrophages differentiation and activity in Adult-onset Still’s disease and COVID-19, two hyperinflammatory diseases sharing similar transcriptomic profiles
Source: Front Immunol. 2023 Apr 21;14:1148268. doi: 10.3389/fimmu.2023.1148268 (PMC10160453; doi:10.3389/fimmu.2023.1148268)

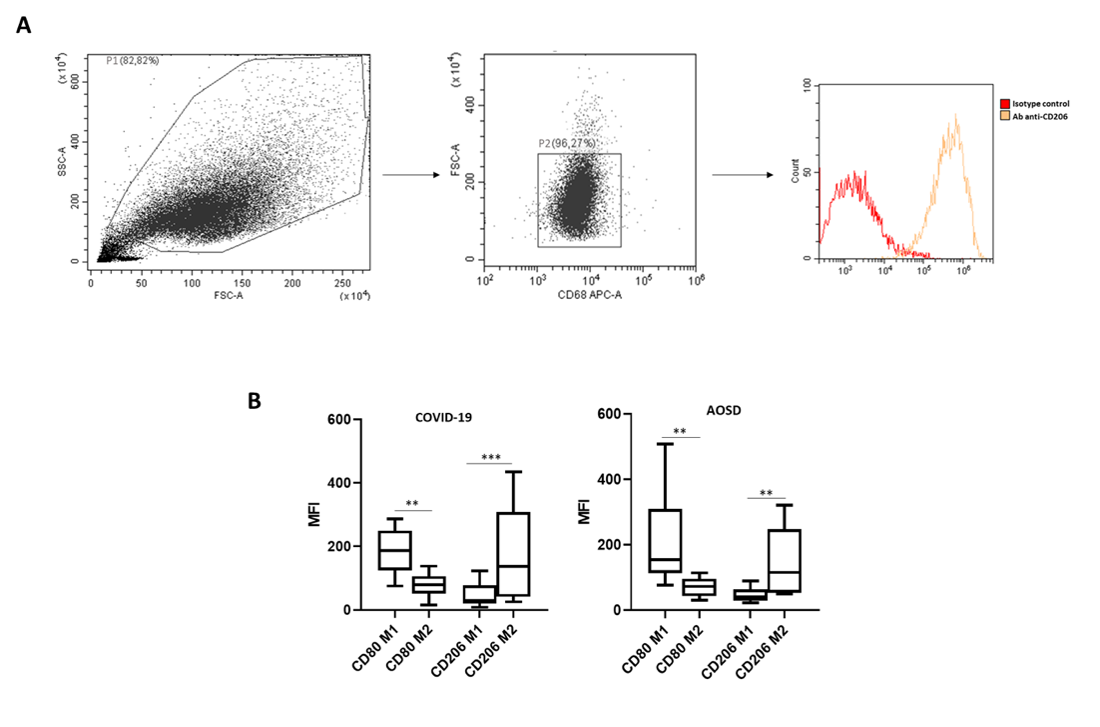

Supplement: Supplementary Figure 1 — (A) Gating strategy for flow cytometry analysis of surface markers in monocyte-derived macrophages. Population was first defined using forward scatter (FSC) and side scatter (SSC). Debris and apoptotic cells were excluded. The expression of surface markers CD206 and CD80 were analyzed on CD68-positive cells. (B) M1 and M2 Macrophages Polarization. Analysis of CD80 and CD206 expression, reported as mean fluorescence intensity, in monocyte-derived macrophages polarized towards M1 and M2 phenotype. MFI= mean fluorescence intensity. [file Image_1.png]

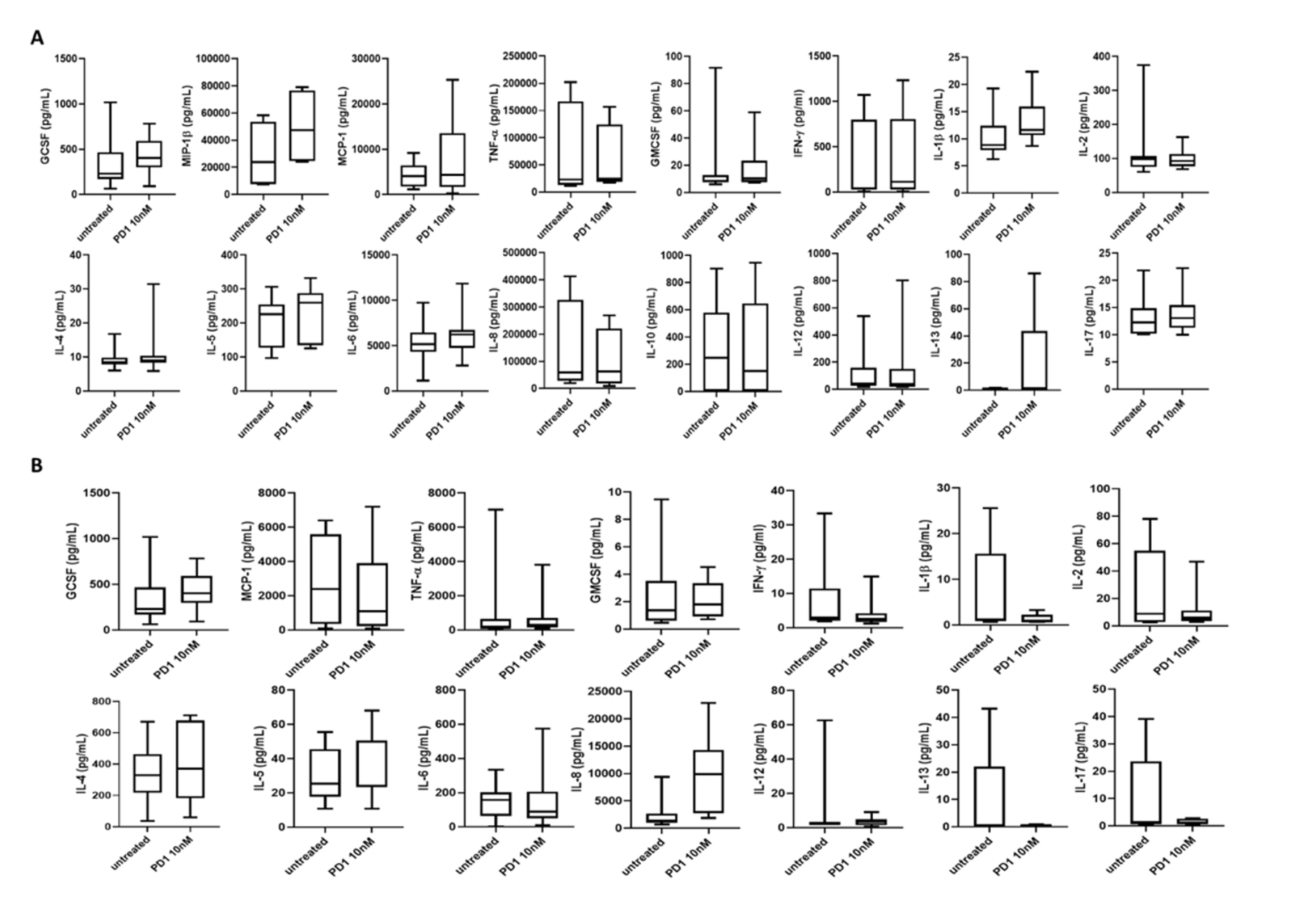

Supplement: Supplementary Figure 2 — Cytokines and chemokines levels in supernatants of M1 (A) and M2 (B) macrophages from COVID-19 patients treated in vitro with PD1. Data are reported as pg/mL and represent 12 independent experiments. [file Image_2.jpeg]

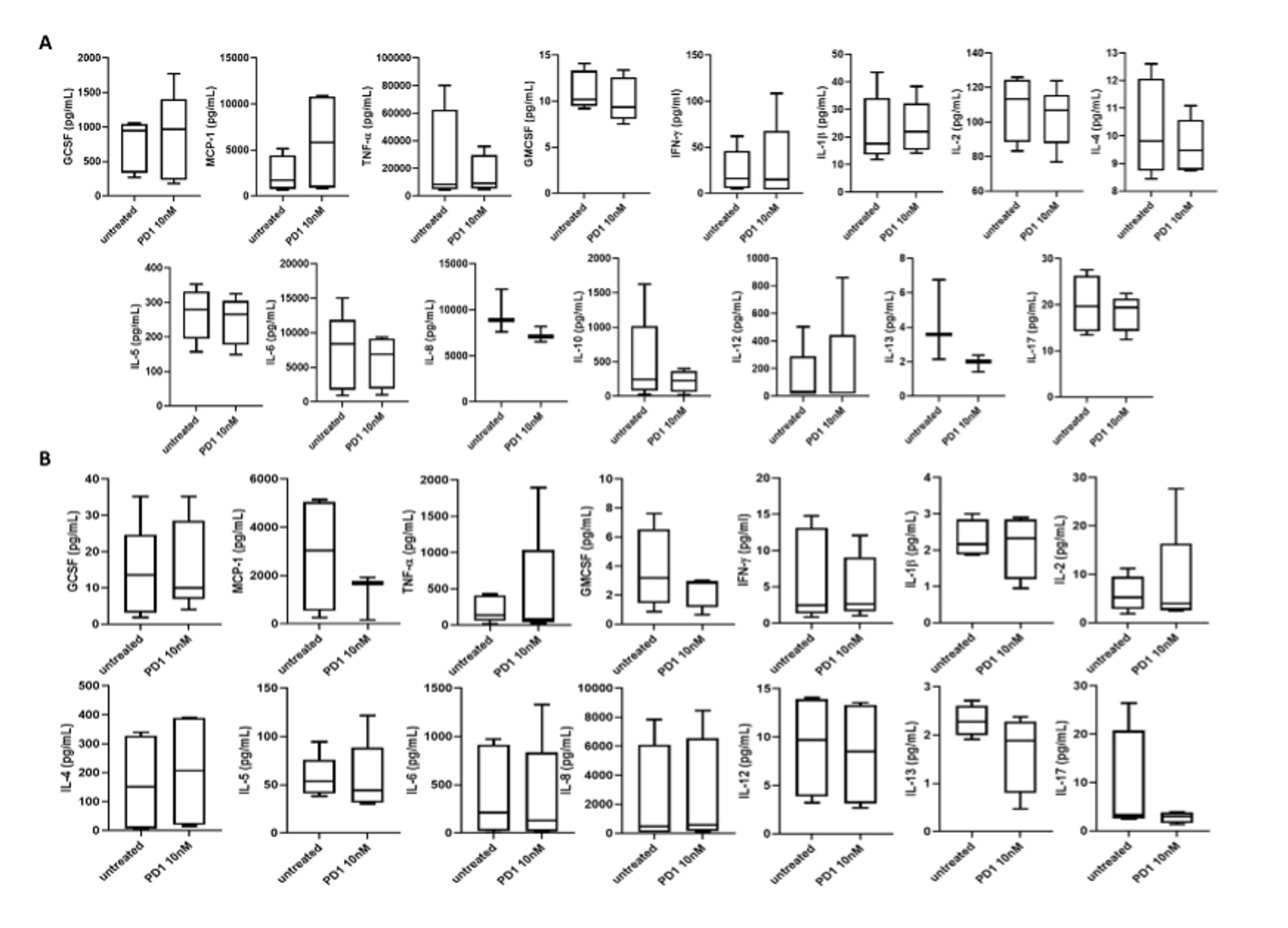

Supplement: Supplementary Figure 3 — Cytokines and chemokines levels in supernatants of M1 (A) and M2 (B) macrophages from AOSD patients treated in vitro with PD1. Data are reported as pg/mL and represent 10 independent experiments. [file Image_3.jpeg]

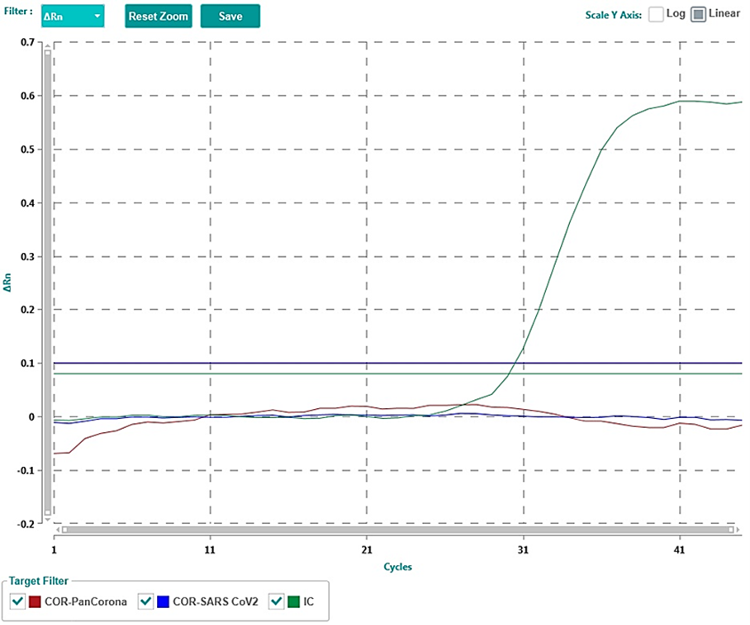

Supplement: Supplementary Figure 4 — Representative assay amplification curves of SARS-CoV-2 RNA of one sample obtained from in vitro experiments on monocytes-derived macrophages from COVID-19 patients. Green line represents the amplification internal control, red line represents the amplification for SARS-CoV2 E (Envelope) gene while the blue line shows the amplification for SARS-CoV2 S (Spike) gene. [file Image_4.png]
